# AKT-GSK3*β* Signaling Pathway Regulates Mitochondrial Dysfunction-Associated OPA1 Cleavage Contributing to Osteoblast Apoptosis: Preventative Effects of Hydroxytyrosol

**DOI:** 10.1155/2019/4101738

**Published:** 2019-06-02

**Authors:** W. J. Cai, Y. Chen, L. X. Shi, H. R. Cheng, I. Banda, Y. H. Ji, Y. T. Wang, X. M. Li, Y. X. Mao, D. F. Zhang, P. P. Dai, X. Y. Sun, X. H. Ning, S. B. Huang, J. F. Ma, S. F. Zhao

**Affiliations:** ^1^Institute of Stomatology, School and Hospital of Stomatology, Wenzhou Medical University, Wenzhou, China; ^2^Department of Prosthodontics, School and Hospital of Stomatology, Wenzhou Medical University, Wenzhou, China; ^3^Department of Stomatology, Taizhou Hospital, Wenzhou Medical University, Linhai, China; ^4^Department of Periodontics, School and Hospital of Stomatology, Wenzhou Medical University, Wenzhou, China; ^5^Department of Oral Maxillofacial Surgery, School and Hospital of Stomatology, Wenzhou Medical University, Wenzhou, China

## Abstract

Oxidative stress (OS) induces osteoblast apoptosis, which plays a crucial role in the initiation and progression of osteoporosis. Although OS is closely associated with mitochondrial dysfunction, detailed mitochondrial mechanisms underlying OS-induced osteoblast apoptosis have not been thoroughly elucidated to date. In the present study, we found that mitochondrial abnormalities largely contributed to OS-induced osteoblast apoptosis, as evidenced by enhanced production of mitochondrial reactive oxygen species; considerable reduction in mitochondrial respiratory chain complex activity, mitochondrial membrane potential, and adenosine triphosphate production; abnormality in mitochondrial morphology; and alteration of mitochondrial dynamics. These mitochondrial abnormalities were primarily mediated by an imbalance in mitochondrial fusion and fission through a protein kinase B- (AKT-) glycogen synthase kinase 3*β-* (GSK3*β-*) optic atrophy 1- (OPA1-) dependent mechanism. Hydroxytyrosol (3,4-dihydroxyphenylethanol (HT)), an important compound in virgin olive oil, significantly prevented OS-induced osteoblast apoptosis. Specifically, HT inhibited OS-induced mitochondrial dysfunction by decreasing OPA1 cleavage and by increasing AKT and GSK3*β* phosphorylation. Together, our results indicate that the AKT-GSK3*β* signaling pathway regulates mitochondrial dysfunction-associated OPA1 cleavage, which may contribute to OS-induced osteoblast apoptosis. Moreover, our results suggest that HT could be an effective nutrient for preventing osteoporosis development.

## 1. Introduction

Osteoporosis is characterized by decreased bone mass and increased fracture susceptibility [1]. Because osteoporosis increases the risk of fragile bone fractures, several therapies have been developed for preventing and treating osteoporosis. However, effective therapeutic strategies for preventing and treating osteoporosis are unavailable because of the limited understanding of mechanisms underlying this disease [2].

Osteoblast apoptosis plays a crucial role in bone development and maintenance and partly contributes to osteoporosis in the presence of sex steroid deficiency, glucocorticoid excess, and aging [3]. Oxidative stress (OS) is characterized by the overproduction of reactive oxygen species (ROS) because of a prooxidant/antioxidant imbalance [4]. Studies have shown that increased OS is observed in osteoporosis and might be a major cause of osteoblast apoptosis [5–8]. Therefore, alleviation of OS-induced osteoblast apoptosis is important for preventing or at least delaying the loss of bone mass in osteoporosis.

The mitochondria are a major source and the principal target of ROS [9]. Mitochondrial dysfunction affects osteoblast function [10] and is a key mechanism underlying OS-induced osteoblast apoptosis [11]. Therefore, modulation of mitochondrial function may be a novel therapeutic strategy to prevent osteoblast apoptosis. Furthermore, the mitochondria are dynamic organelles that undergo continuous fission and fusion. Previous studies have shown that ROS production is correlated with decreased mitochondrial fusion [12, 13]. These findings indicate that OS induces mitochondrial dysfunction and that regulating mitochondrial fusion may be a strategy to prevent OS-associated bone disorders.

Optic atrophy 1 (OPA1) is required for the fusion of mitochondrial inner membranes [14] and exists in two forms, a long form (L-OPA1) and a short form (S-OPA1) [15]. These two OPA1 forms are necessary for generating fusion-competent mitochondria. Mitochondrial ROS (mtROS) production and its associated dysfunction are closely correlated with mitochondrial dynamics disruption. Both the inhibition of adenosine triphosphate (ATP) synthase and loss of mitochondrial membrane potential (MMP) are the crucial stimuli that regulate OPA1 processing [16–18]. Moreover, OPA1-modulated mitochondrial fusion is vital for preventing fibroblast apoptosis and for maintaining mitochondrial function through various mechanisms [15, 19–21]. However, it is unclear whether OPA1-mediated mitochondrial events regulate OS-induced osteoblast apoptosis.

Protein kinase B (AKT), a serine/threonine protein kinase, is a critical regulator of cell survival and proliferation. We previously confirmed that AKT is rapidly activated in response to OS and phosphorylates glycogen synthase kinase 3*β* (GSK3*β*) at Ser9. The AKT-GSK3*β* signaling pathway plays a significant role in regulating OS-induced osteoblast apoptosis and mitochondrial dysfunction [11]. Moreover, a recent study has suggested that OPA1-dependent mitochondrial function is mediated by the AKT-mammalian target of rapamycin- (mTOR-) nuclear factor kappa-B (NF*κ*B) pathway [22]. Furthermore, GSK3*β* plays a vital regulatory role in altering mitochondrial dynamics [13]. Therefore, we hypothesized that OPA1 is regulated by the AKT-GSK3*β* pathway and contributes to OS-induced osteoblast apoptosis. However, the involvement of this pathway in osteoblast apoptosis needs to be verified.

Hydroxytyrosol (3,4-dihydroxyphenylethanol (HT)), the most active polyphenolic compound in olive oil and a potent scavenger of several free radical species, exerts protective effects against OS in Jurkat cells [23, 24]. An *in vivo* study showed that olive fruits, rich in micronutrients, might decrease bone loss caused by ovariectomy and talc granulomatosis in rats [25]. Moreover, HT exerts an antiosteoporosis effect by inhibiting multinucleated osteoclast formation and by promoting calcium deposition in osteoblasts [26]. However, it is unclear whether HT maintains osteoblast survival by preventing OS-induced apoptosis. Studies have reported that HT inhibits cardiomyocyte and primary rat Sertoli cell apoptosis through the phosphatidylinositol 3 kinase- (PI3K-) AKT signaling pathway [22, 27]. Interestingly, Wang et al. reported that HT acetate directly targeted an OPA1-dependent mitochondrial pathway to prevent muscle degeneration [13]. Despite the determination of the different pharmacological effects of HT, it is unclear whether HT exerts a protective effect against OS-induced osteoblast apoptosis by preventing OPA1 cleavage and by activating the AKT-GSK3*β* signaling pathway.

Therefore, the present study investigated (1) the involvement of OPA1-regulated mitochondrial events in OS-induced osteoblast apoptosis, (2) the relationship between the AKT-GSK3*β* signaling pathway and OPA1, (3) the cytoprotective potential of HT against OS-induced osteoblast apoptosis, and (4) mechanisms underlying the protective effects of HT.

## 2. Materials and Methods

### 2.1. Reagents

Cell culture medium and supplements were purchased from Life Technologies (Grand Island, NY, USA). Antibodies against phosphorylated AKT (Ser473) (p-AKT) (#4060S), AKT (#2920S), phosphorylated GSK3*β* (Ser9) (p-GSK3*β*) (#9336S), GSK3*β* (#9832S), and *β*-actin (#3700) were obtained from Cell Signaling Technology (Beverly, MA, USA), and antibody against OPA1 (#612606) was obtained from BD Biosciences (San Jose, CA, USA). Chamber slides, goat anti-rabbit (#656120) and anti-mouse (#626520) secondary antibodies, Lipofectamine 3000 (#L3000015), and 4′,6-diamidino-2-phenylindole (DAPI) (#P36931) were obtained from Invitrogen (Carlsbad, CA, USA). MitoSOX Red (#M36008), tetramethylrhodamine methyl ester (TMRM) (#T668), MitoTracker Green (MT Green) (#M7514), and MitoTracker Deep Red (#M22426) (all from Molecular Probes, USA) were purchased from Life Technologies. Terminal deoxynucleotidyl transferase dUTP nick end labeling (TUNEL) assay kits (#11684795910) were obtained from Roche (Mannheim, Germany). HT (#H4291), H_2_O_2_ (#88597), 4-benzyl-2-methyl-1,2,4-thiadiazolidine-3,5-dione (TDZD-8) (#T8325), LY294002 (#L9908), *N*-acetyl-l-cysteine (NAC) (#A7250), and 3-(4,5-dimethylthiazol-2-yl)-2,5-diphenyltetrazolium bromide (MTT) (#M2128) were obtained from Sigma-Aldrich (St. Louis, MO, USA). ATP assay kit (#S0026) was obtained from Beyotime Institute of Biotechnology (Shanghai, China).

### 2.2. Cell Culture

The murine osteoblast MC3T3-E1 subclone 14 line used in this study was obtained from the American Type Culture Collection (Manassas, VA, USA). The cells were maintained in an *α*-modified minimal essential medium supplemented with 10% fetal bovine serum (FBS) and antibiotics (100 IU/mL penicillin G and 100 ng/mL streptomycin) at 37°C in a humidified incubator equilibrated with 5% CO_2_ and 95% air. The culture medium was replenished twice per week. We followed the methods of Mao et al. [8].

### 2.3. Cell Treatment

Test compounds were prepared as stock solutions and were diluted to the desired final concentrations immediately before use. Working concentrations of the test compounds were as follows: 0.1, 0.25, 0.5, 0.75, and 1.0 mM for H_2_O_2_ and 5, 10, 20, 50, and 100 *μ*M for HT. Concentrations of TDZD-8 (5 *μ*M), LY294002 (10 *μ*M), and NAC (2.5 mM) were used according to those mentioned in previous studies [11, 28]. The final concentration of dimethyl sulfoxide (DMSO) in cell culture was <0.5% in all experiments. The cells were treated with or without H_2_O_2_ and the indicated test compounds for different time periods, according to the experimental protocol.

### 2.4. RNA Interference and Cell Transfection

A specific small interfering RNA (siRNA) against the OPA1 gene was purchased from RiboBio Co. Ltd. (Guangdong, China). The MC3T3-E1 cells grown in 24-well plates were transfected with 50 nM OPA1 siRNA or negative control siRNA by using Lipofectamine 3000 reagent in serum-free Opti-MEM, according to the manufacturer's instructions, and were harvested at 48 h after the transfection.

### 2.5. Cell Viability Assay

Cell viability was determined by performing MTT colorimetric assay. The MC3T3-E1 cells were plated in 96-well plates (density, 1 × 10^4^ cells/well) and were exposed to H_2_O_2_ in the absence or presence of the other test compounds. After incubation, the cells were washed twice with phosphate-buffered saline (PBS) and were incubated in an FBS-free medium (100 *μ*L/well) supplemented with 10 *μ*L MTT solution (5 mg/mL) at 37°C. After 4 h, culture supernatant was removed and resulting formazan crystals were dissolved in 150 *μ*L DMSO for 20 min. Next, the plates were agitated for 15 s and absorbance was measured at 570 nm by using a microplate reader.

### 2.6. Measurement of Apoptosis by Performing Flow Cytometry and TUNEL Assay

Osteoblast apoptosis was determined using FITC-labeled Annexin-V. Cell necrosis was determined using 1 *μ*g/mL propidium iodide (PI). After exposure to the various experimental conditions, the cells were trypsinized and labeled with the fluorochromes at 37°C. Cytofluorometric analysis was performed using an FACS scanner (Becton Dickinson, NY, USA).

TUNEL assay was performed to identify the rate of cell apoptosis. For performing the TUNEL assay, the cells were inoculated on a coverslip, fixed with 4% paraformaldehyde in PBS, and permeabilized with 0.2% Triton X-100 in citrate buffer. Next, the cells were incubated with TUNEL reaction mixture at 37°C for 1 h and were counterstained with DAPI. Apoptotic cells were observed by a technician who was blinded to the treatments by using a fluorescence microscope (Leica TCS SPE, Germany). The percentage of apoptotic cells was estimated by counting a total of 300 cells from random fields. We followed the methods of Mao et al. [8].

### 2.7. Western Blotting Analysis

The cells were incubated under the different experimental conditions indicated above, collected, and homogenized in a cell lysis buffer (Cell Signaling Technology). Protein concentrations in the cell lysates were determined using the Bradford protein assay kit (Thermo Fisher Scientific, USA). Next, proteins present in the cell lysates were separated by performing sodium dodecyl sulfate-polyacrylamide gel electrophoresis on a 12% gel and were transferred onto polyvinylidene difluoride (PVDF) membranes (Bio-Rad, Hercules, CA, USA). The PVDF membranes were blocked with 5% nonfat dry milk diluted in Tris-buffered saline (pH 7.4) containing 0.05% Tween-20 (TBST) and were mildly agitated on a shaker for 90 min at room temperature. Next, the membranes were washed two times with TBST (3 min per wash) and were incubated overnight at 4°C with the indicated primary antibodies against p-AKT (dilution, 1 : 2000), AKT (dilution, 1 : 2000), p-GSK3*β* (dilution, 1 : 2000), GSK3*β* (dilution, 1 : 2000), OPA1 (dilution, 1 : 2000), and *β*-actin (dilution, 1 : 8000). Next, the membranes were washed three times with TBST (5 min per wash) and were incubated with the anti-mouse or anti-rabbit secondary antibody (dilution, 1 : 5000) in 5% nonfat dry milk diluted in TBST for 60 min at room temperature. Finally, the membranes were washed three times with TBST (5 min per wash) and protein bands were visualized using an enhanced chemiluminescence detection kit (Thermo Fisher Scientific). Quantitative densitometric analysis of the identified protein bands was performed using an imaging system (Bio-Rad), and quantification was performed using NIH ImageJ software (available in the public domain).

### 2.8. Functional Imaging Assays

The MC3T3-E1 cells were seeded in chamber slides at a density of 10^4^ cells/well and were treated with H_2_O_2_ and the other test compounds. MitoSOX, a unique fluorogenic dye, was used to selectively detect superoxide production in the mitochondria of live cells. The cells were incubated in a fresh culture medium containing 2.5 *μ*M MitoSOX for 30 min. MMP was assessed by treating the cells with H_2_O_2_ and the other test compounds, followed by costaining with MT Green (100 nM) and TMRM (100 nM) for 30 min, similar to that performed in our previous study [11].

The mitochondria were incubated with 100 nM MitoTracker Red for 30 min at 37°C and were fixed to visualize their morphology. Images were captured by a technician who was blinded to the treatments by using a confocal microscope (Leica TCS SPE). The excitation wavelength for MitoSOX, TMRM, or MitoTracker Red was 543 nm and that for MT Green was 488 nm. The obtained images were processed using MetaMorph (Molecular Devices), and fluorescence signals corresponding to mitochondrial length and area were quantified using the NIH ImageJ software. Mitochondrial size, density, and fluorescence intensity were quantified by an investigator who was blinded to the experimental groups. The experiment was performed in triplicate, and >100 clearly identifiable mitochondria from 10–15 randomly selected cells were measured in each per experiment [29].

### 2.9. Mitochondrial Complex Activity Assays

Activities of NADH-ubiquinone reductase (complex I), succinate-CoQ oxidoreductase (complex II), CoQ-cytochrome c reductase (complex III), cytochrome c oxidase (complex IV), and ATP synthase (complex V) were measured spectrophotometrically by performing conventional assays, according to protocols reported in previous studies [30, 31]. The activities of all the mitochondrial complexes were adjusted using the expression level of each complex, and all experiments were performed in triplicate.

### 2.10. Measurement of ATP Production

For measuring ATP levels, whole-cell lysates were prepared by lysing the cells in a lysis buffer provided in the ATP assay kit. After centrifugation at 12,000 × *g* and 4°C for 5 min, supernatants obtained were transferred to a new 1.5 mL tube for analyzing ATP production. Luminescence of a 100 *μ*L sample was assayed using a luminometer (Molecular Devices) and 100 *μ*L ATP detection buffer. A standard curve of ATP concentrations (1 nM to 1 *μ*M) was prepared from a known amount. All experiments were performed in triplicate, which was consistent with our previous study [8].

### 2.11. Data Analysis

Data are presented as mean ± SD. All statistical analyses were performed using StatView software (version 5.0.1; SAS Institute). Comparisons between multiple groups were performed using one-way ANOVA followed by the post hoc Fisher test, where applicable. A *p* value of <0.05 was considered to be statistically significant.

## 3. Results

### 3.1. OS Induces Apoptosis and Mitochondrial Dysfunction in the MC3T3-E1 Cells

**Figure 1:**
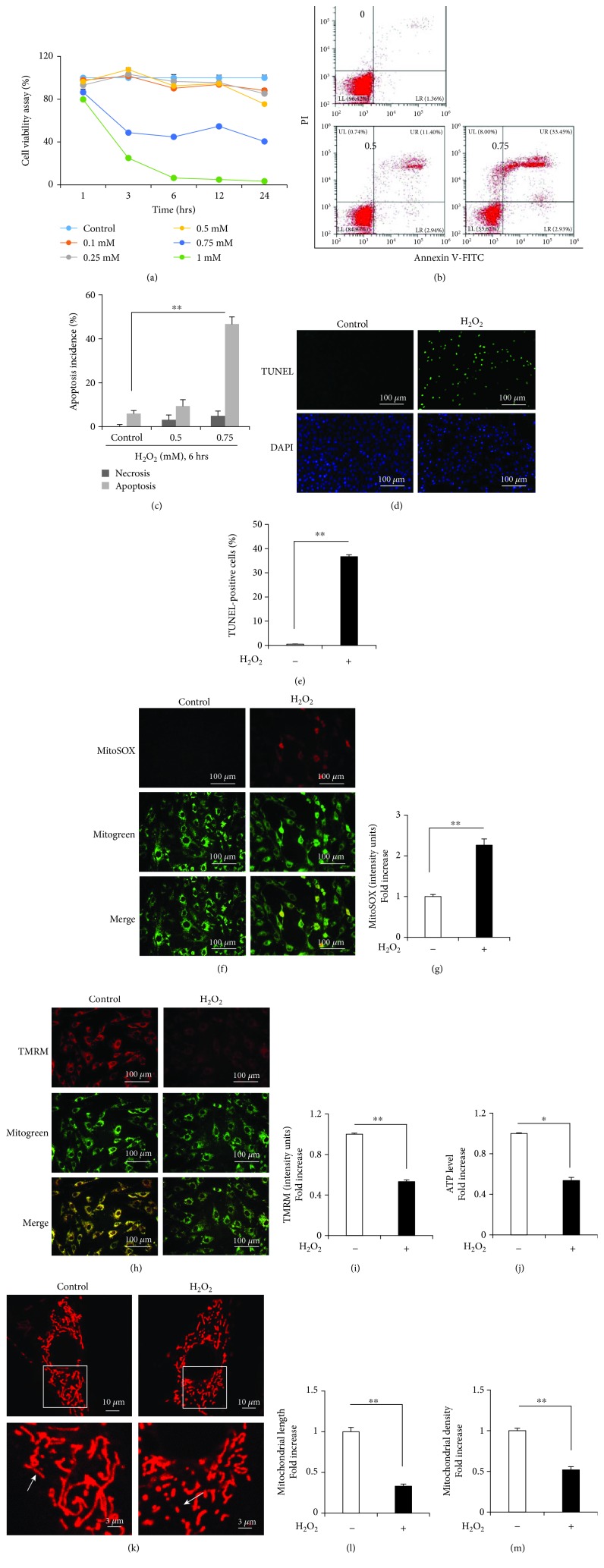
H_2_O_2_ induces the apoptosis of and mitochondrial dysfunction in the MC3T3-E1 cells. (a) Cell viability was determined by performing the MTT assay. Error bars indicate SD (*n* = 6). (b, c) Flow cytometry analysis of cell apoptosis. Error bars indicate SD (*n* = 6). (d, e) TUNEL assay was performed to determine the rate of cell apoptosis. H_2_O_2_ induced mitochondrial dysfunction in osteoblasts. Error bars indicate SD (*n* = 300); scale bars, 100 *μ*m. (f, g) Representative images showing MitoSOX staining and quantification in the indicated groups; scale bar, 100 *μ*m. (h, i) Representative images showing TMRM staining and quantification in the indicated groups. MT Green staining was performed to show the mitochondria; scale bar, 100 *μ*m. (j) ATP levels were determined in the presence or absence of H_2_O_2_. (k–m) Representative images showing mitochondrial morphology, length, and density in the indicated groups. Error bars indicate SD (*n* = 3); scale bars, 10 *μ*m. ^∗^ *p* < 0.05 and ^∗∗^ *p* < 0.0001, as determined by comparing selected pairs (each test was repeated three times).

The MC3T3-E1 cells were incubated with 0.1–1 mM H_2_O_2_ for 1, 3, 6, 12, and 24 h. H_2_O_2_ treatment decreased the number of viable cells in a time- and dose-dependent manner (Figure 1(a)). Subsequent experiments were performed by treating the MC3T3-E1 cells with 0.75 mM H_2_O_2_ for 6 h. The Annexin-V/PI dual staining assay showed a dose-dependent increase in the apoptosis of the MC3T3-E1 cells (Figures 1(b) and 1(c)). The proapoptotic effects of H_2_O_2_ were further verified by performing the TUNEL assay (Figure 1(d)). The percentage of TUNEL-positive cells significantly increased from 0% to 35% after treatment with 0.75 mM H_2_O_2_ (Figure 1(e)). Moreover, H_2_O_2_ treatment dramatically increased mtROS production (indicated by MitoSOX staining; Figures 1(f) and 1(g)) and decreased MMP (indicated by TMRM staining; Figures 1(h) and 1(i)) compared with that in control cells. In addition, H_2_O_2_ treatment significantly reduced ATP levels in the MC3T3-E1 cells (Figure 1(j)). MitoTracker Red staining showed that the mitochondria of the H_2_O_2_-treated MC3T3-E1 cells were fragmented, misshapen, bleb like, and collapsed, indicating that H_2_O_2_-induced OS promoted mitochondrial fission that decreased the length and density of the mitochondria in the MC3T3-E1 cells (Figures 1(k)–1(m)).

### 3.2. HT Exerts a Protective Effect against OS-Induced Mitochondria-Related Events and Osteoblast Apoptosis

**Figure 2:**
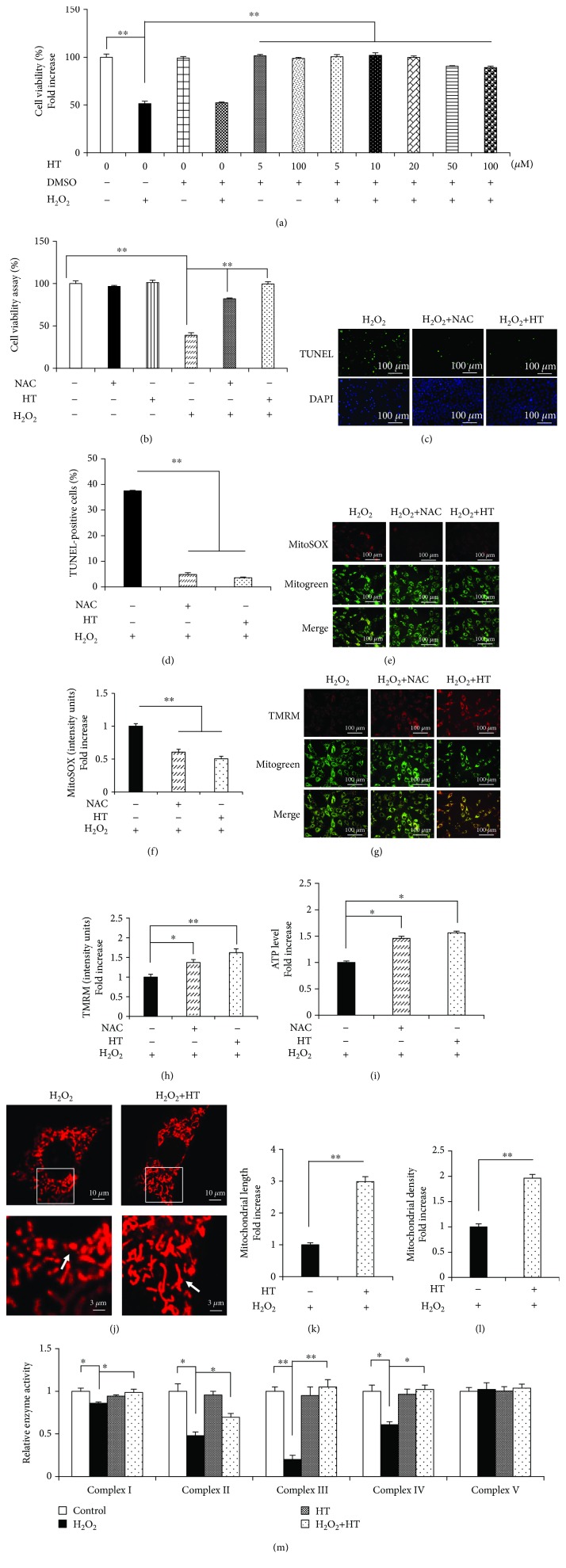
HT prevents osteoblast apoptosis and mitochondrial dysfunction. (a) Cell viability was determined by performing the MTT assay. Error bars indicate SD (*n* = 6). (b) Cell viability was determined based on MTT reduction in the osteoblasts treated with (+) or without (-) HT in the presence of H_2_O_2_ (+) or NAC (+). Error bars indicate SD (*n* = 6). (c, d) TUNEL assay was performed to determine the rate of cell apoptosis. Error bars indicate SD (*n* = 300); scale bars, 100 *μ*m. (e, f) Representative images showing MitoSOX staining and quantification in the indicated groups; scale bar, 100 *μ*m. (g, h) Representative images showing TMRM staining and quantification in the indicated groups. MT Green staining was performed to show the mitochondria; scale bar, 100 *μ*m. (i) ATP levels were determined in the presence or absence of HT or NAC. (j–l) Representative images showing mitochondrial morphology, length, and density in the indicated groups. Error bars indicate SD (*n* = 3); scale bars, 10 *μ*m. (m) Activity of the mitochondrial complexes in the indicated groups. Error bars indicate SD (*n* = 6). ^∗^ *p* < 0.05 and ^∗∗^ *p* < 0.0001, as determined by comparing selected pairs (each test was repeated three times).

HT exerts protective effects against osteoporosis; however, the underlying molecular mechanism is unclear. To investigate whether HT ameliorated OS-induced osteoblast apoptosis, the MC3T3-E1 cells were treated with different HT concentrations, followed by treatment with H_2_O_2_. Treatment with 5 *μ*M HT for 1 h exerted a remarkable protective effect on the viability of the MC3T3-E1 cells without exerting any cytotoxic effects (Figure 2(a)) Therefore, 5 *μ*M HT was chosen as the HT concentration for performing subsequent experiments. Furthermore, we found that HT showed the highest protective activity of 30% as compared to H_2_O_2_ alone, as confirmed by the results of the TUNEL assay (Figures 2(c) and 2(d)). These data indicate that HT attenuates H_2_O_2_-induced apoptosis of the MC3T3-E1 cells.

Moreover, we found that HT significantly ameliorated mtROS generation (Figures 2(e) and 2(f)), restored MMP (Figures 2(g) and 2(h)), and increased ATP production (Figure 2(i)). Next, we examined whether the different antioxidant treatments examined in the present study prevented H_2_O_2_-induced osteoblast mitochondrial dysfunction. The MC3T3-E1 cells treated with NAC and H_2_O_2_ showed considerably improved viability and decreased apoptosis compared with the cells treated with H_2_O_2_ alone (Figures 2(b)–2(d)). In addition, similar to HT, NAC markedly decreased mtROS levels (Figures 2(e) and 2(f)) and increased MMP and ATP levels (Figures 2(g)–2(i)), suggesting that HT exerted antioxidant effects similar to NAC. Furthermore, HT efficiently abolished OS-induced alterations in mitochondrial morphology, as indicated by an increase in mitochondrial length and density (Figures 2(j)–2(l)). Mitochondrial complexes are essential for mitochondrial function. Therefore, we evaluated the key enzymes associated with the respiratory chain in the present study. We found that H_2_O_2_ decreased the activities of the mitochondrial complexes I, II, III, and IV and that HT treatment restored the activities of these complexes (Figure 2(m)). Collectively, these results indicate that HT exerts efficient antioxidant and mitochondria-protective effects and protects against OS-induced osteoblast apoptosis.

### 3.3. HT Prevents OS-Induced Osteoblast Apoptosis by Reducing OPA1 Cleavage

**Figure 3:**
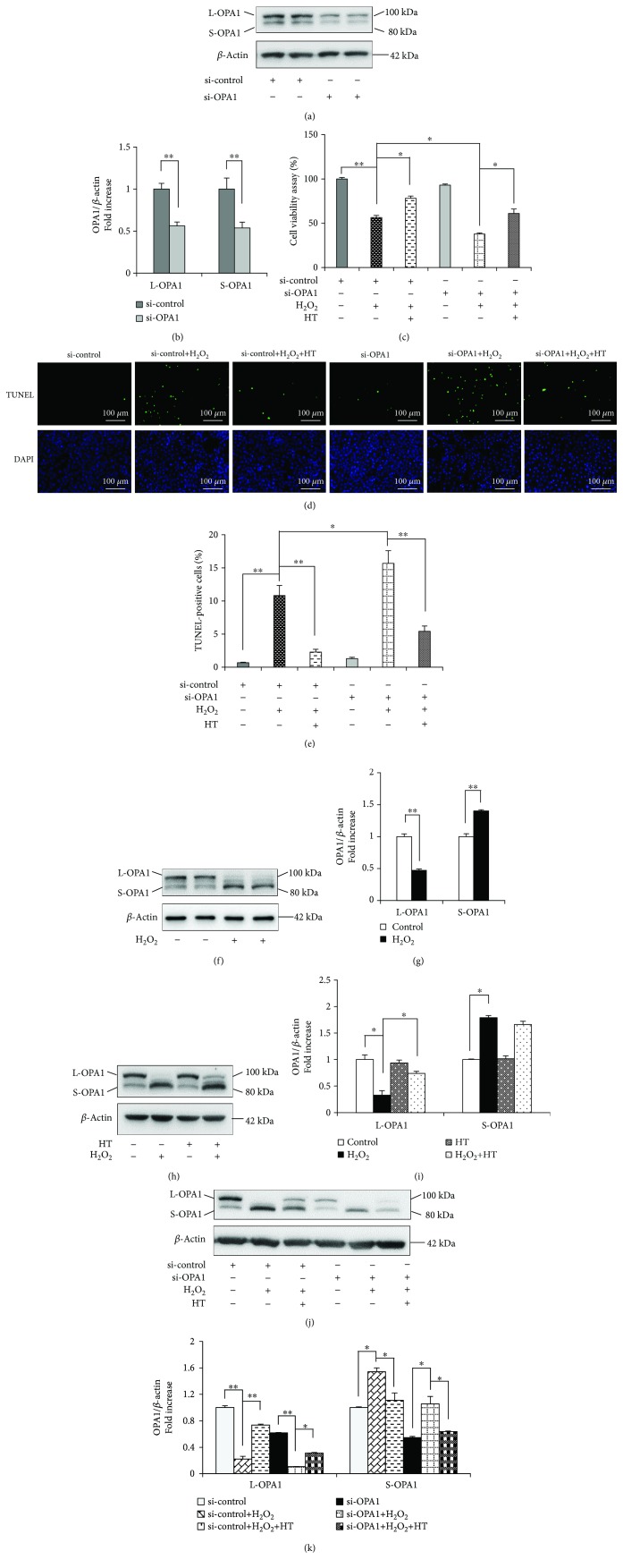
HT prevents OS by reducing OPA1 cleavage. (a, b) Representative immunoreactive bands and relative OPA1 levels in the osteoblasts transfected with 50 nM OPA1 siRNA or negative control siRNA. Error bars indicate SD (*n* = 6). (c) Cell viability was determined based on MTT reduction in the osteoblasts treated with (+) or without (-) HT in the presence of H_2_O_2_ (+). Error bars indicate SD (*n* = 6). (d, e) TUNEL assay was performed to identify the rate of cell apoptosis. Error bars indicate SD (*n* = 300); scale bars, 100 *μ*m. (f, g) Representative immunoreactive bands of OPA1 in the osteoblasts treated with (+) or without (-) H_2_O_2_. Quantification of the immunoreactive bands of OPA1 relative to that of *β*-actin. Error bars indicate SD (*n* = 6). (h, i) Representative immunoreactive bands and relative OPA1 levels in the osteoblasts treated with (+) or without (-) HT in the presence (+) or absence (-) of H_2_O_2_ (+). (j) Representative immunoreactive bands of OPA1 in the indicated groups. (k) Quantification of the immunoreactive bands of OPA1 relative to that of *β*-actin. Error bars indicate SD (*n* = 6). Full-length blots are presented in Supplementary Figure 1. ^∗^ *p* < 0.05 and ^∗∗^ *p* < 0.0001, as determined by comparing selected pairs (each test was repeated three times).

OPA1 is one of the most important mitochondrial dynamics proteins. The role of OPA1 in the MC3T3-E1 cells was investigated using a specific OPA1 siRNA. The cells transfected with the OPA1 siRNA showed significantly lower L-OPA1 and S-OPA1 levels than the cells transfected with the negative control siRNA (Figures 3(a) and 3(b)). Previous studies have reported that OPA1 loss does not induce cytochrome c release from the mitochondria or cell death but increases apoptotic susceptibility [24]. The lowest viability and the highest apoptosis rate were detected in the MC3T3-E1 cells treated with the OPA1 siRNA and H_2_O_2_, as confirmed by performing the MTT assay (Figure 3(c)) and TUNEL assay (Figures 3(d) and 3(e)). We observed that H_2_O_2_ treatment remarkably reduced the L-OPA1 level and increased the S-OPA1 level (Figures 3(f) and 3(g)) and that these levels were restored after HT treatment (Figures 3(h) and 3(i)). Moreover, the transfection of the MC3T3-E1 cells with the OPA1 siRNA abolished the protective effects of HT (Figures 3(j) and 3(k)). These results suggest that HT prevents OS-induced apoptosis of MC3T3-E1 cells by reducing OPA1 cleavage.

### 3.4. OPA1 Knockdown Abolishes the Protective Effects of HT on Mitochondrial Function

**Figure 4:**
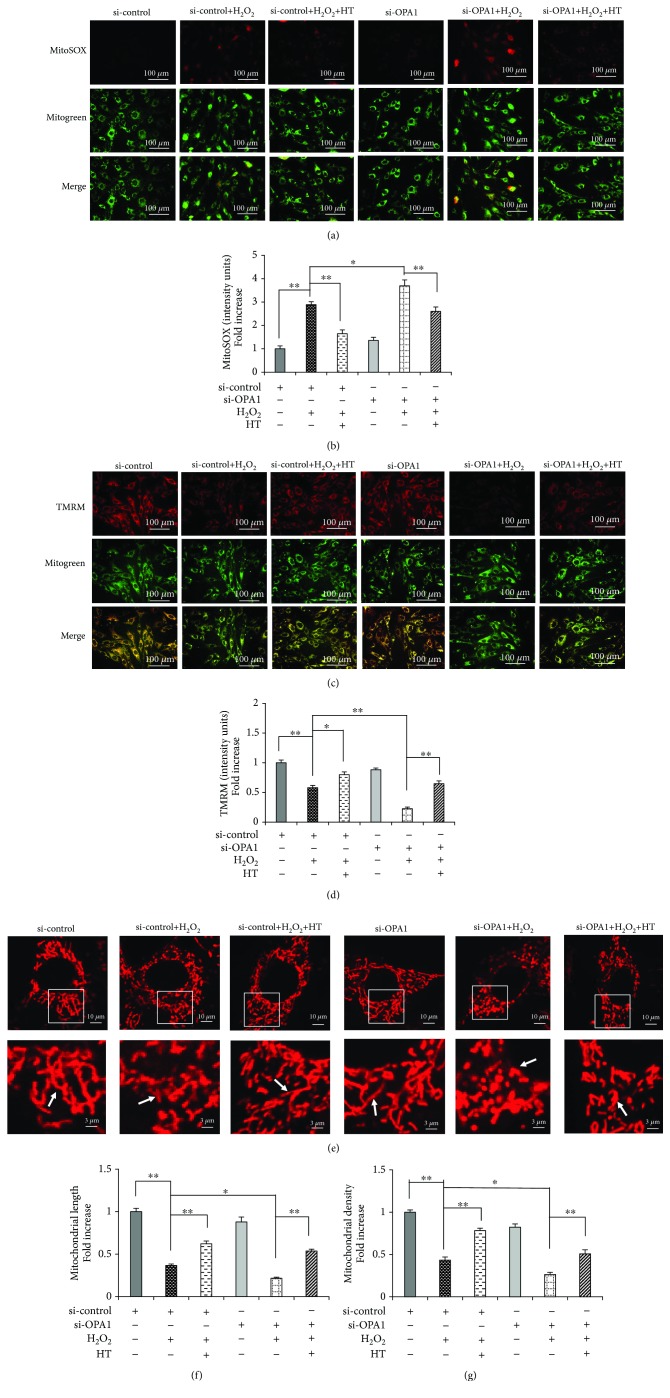
OPA1 knockdown abolishes the protective effects of HT on mitochondrial morphology and function. (a, b) Representative images showing MitoSOX staining and quantification in the indicated groups; scale bar, 100 *μ*m. (c, d) Representative images showing TMRM staining and quantification in the indicated groups. MT Green staining was performed to show the mitochondria; scale bar, 100 *μ*m. (e–g) Representative images showing mitochondrial morphology, length, and density in the indicated groups. Error bars indicate SD (*n* = 3); scale bars, 10 *μ*m. ^∗^ *p* < 0.05 and ^∗∗^ *p* < 0.0001, as determined by comparing selected pairs (each test was repeated three times).

We next explored whether the protective effects of HT were mediated by the regulation of an OPA1-dependent mitochondrial pathway. We found that transfection of the MC3T3-E1 cells with the OPA1 siRNA aggravated mitochondrial morphological abnormalities and dysfunction, as evidenced by the significant increase in mtROS production (Figures 4(a) and 4(b)) and decrease in MMP (Figures 4(c) and 4(d)). Moreover, transfection of the MC3T3-E1 cells with the OPA1 siRNA significantly decreased the mitochondrial length and density compared with those in the control cells (Figures 4(e)–4(g)). However, HT treatment restored the deterioration of the mitochondrial dynamics and function in the presence of OPA1. In contrast, these protective effects of HT were significantly abolished in the cells transfected with the OPA1 siRNA (Figures 3(j)–3(k) and 4(a)–4(g)). Together, these findings suggest that HT prevents osteoblast apoptosis through an OPA1-dependent mitochondrial pathway.

### 3.5. HT Modulates the AKT-GSK3*β* Signaling Pathway during OS-Induced Osteoblast Apoptosis

**Figure 5:**
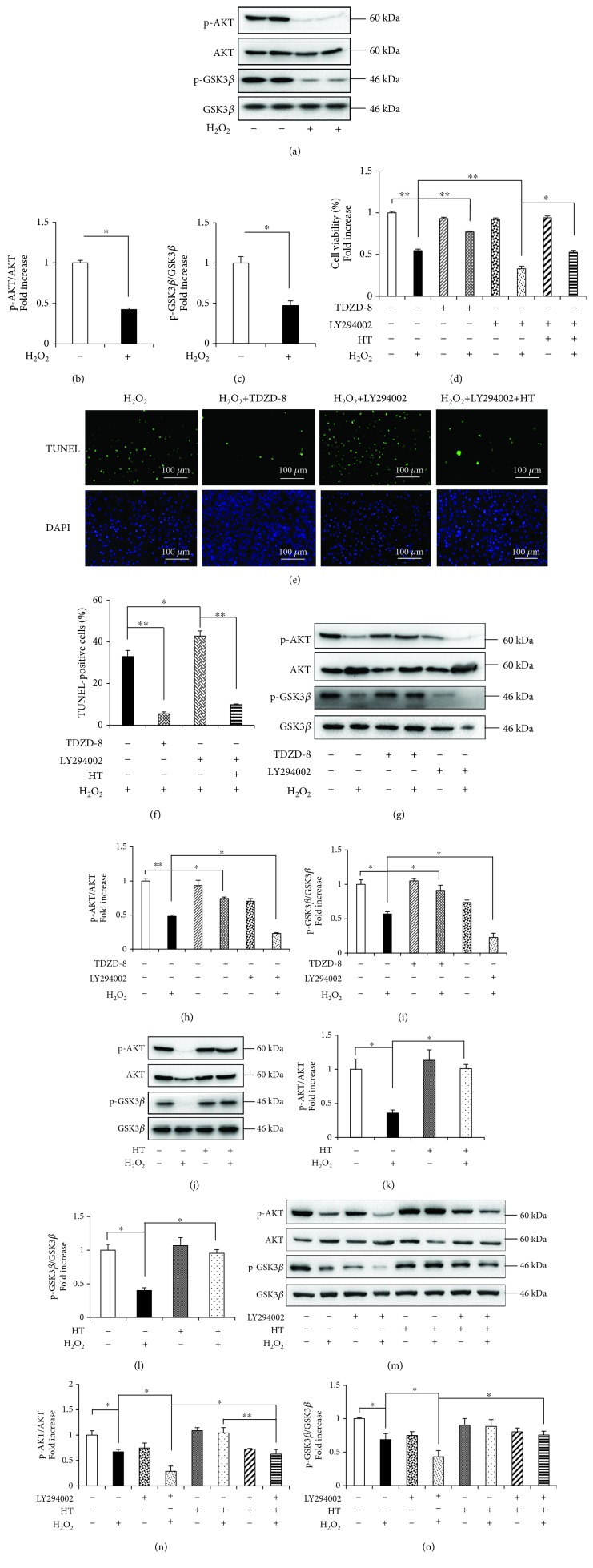
HT protects against H_2_O_2_-induced apoptosis and OS in the MC3T3-E1 cells through the AKT-GSK3*β* signaling pathway. (a–c) Representative immunoblots of p-AKT, AKT, p-GSK3*β*, and GSK3*β* and the relative levels of p-AKT, AKT, p-GSK3*β*, and GSK3*β* in the osteoblasts treated with (+) or without (-) H_2_O_2_. Error bars indicate SD (*n* = 6). (d) Cell viability was determined based on MTT reduction in the osteoblasts treated with (+) or without (-) HT in the presence of H_2_O_2_ (+), LY294002 (+), or TDZD-8 (+). Error bars indicate SD (*n* = 6). (e, f) TUNEL assay was performed to identify the rate of cell apoptosis. Error bars indicate SD (*n* = 300); scale bars, 100 *μ*m. (g–i) Representative immunoreactive bands and relative levels of p-AKT, AKT, p-GSK3*β*, and GSK3*β* in the osteoblasts treated with (+) or without (-) LY294002 or TDZD-8 in the presence (+) or absence (-) of H_2_O_2_. Representative immunoblots are shown at the bottom. Error bars indicate SD (*n* = 6). (j–l) Representative immunoreactive bands and relative levels of p-AKT, AKT, p-GSK3*β*, and GSK3*β* in the osteoblasts treated with (+) or without (-) HT in the presence (+) or absence (-) of H_2_O_2_. (m–o) Densitometric analysis of the immunoreactive bands of p-AKT, AKT, p-GSK3*β*, and GSK3*β* in the osteoblasts treated with (+) or without (-) HT in the presence of H_2_O_2_ (+) or LY294002 (+). Error bars indicate SD (*n* = 6). Full-length blots are presented in Supplementary Figure 1. ^∗^ *p* < 0.05 and ^∗∗^ *p* < 0.0001, as determined by comparing selected pairs (each test was repeated three times).

Our previous study showed that the AKT-GSK3*β* signaling pathway is involved in OS-induced cell apoptosis [11]. In the present study, the H_2_O_2_-treated osteoblasts showed significantly reduced levels of both p-AKT (Ser473) and p-GSK3*β* (Ser9) (Figures 5(a)–5(c)) without any change in the levels of total AKT and GSK3*β*. Treatment with TDZD-8, a pharmaceutical GSK3*β* inhibitor, and LY294002, a PI3K inhibitor, affected H_2_O_2_-induced cytotoxicity and apoptosis, as indicated by the results of the MTT assay (Figure 5(d)) and TUNEL assay (Figures 5(e) and 5(f)), respectively. LY294002 treatment dramatically decreased p-GSK3*β* and p-AKT levels, whereas TDZD-8 only affected the p-GSK3*β* level but did not affect the p-AKT level (Figures 5(g)–5(i)). These results support the hypothesis that AKT regulates GSK3*β* phosphorylation. Moreover, these results indicate that the AKT-GSK3*β* signaling pathway is involved in H_2_O_2_-induced osteoblast apoptosis.

To investigate the mechanism underlying the protective effects of HT against H_2_O_2_-induced osteoblast apoptosis, we analyzed the AKT-GSK3*β* signaling pathway by performing Western blotting analysis. Results of the Western blotting analysis showed that HT increased p-AKT and p-GSK3*β* levels in the presence or absence of H_2_O_2_ (Figures 5(j)–5(l)). However, LY294002 abolished the antiapoptotic effect of HT (Figures 5(m)–5(o)). These results suggest that HT sufficiently promotes AKT activation and inhibits GSK3*β* activity under OS. Therefore, we concluded that HT prevented osteoblast apoptosis by directly modulating the AKT-GSK3*β* signaling pathway.

### 3.6. HT Exerts a Protective Effect on Mitochondrial Morphology and Function through the AKT-GSK3*β* Signaling Pathway

**Figure 6:**
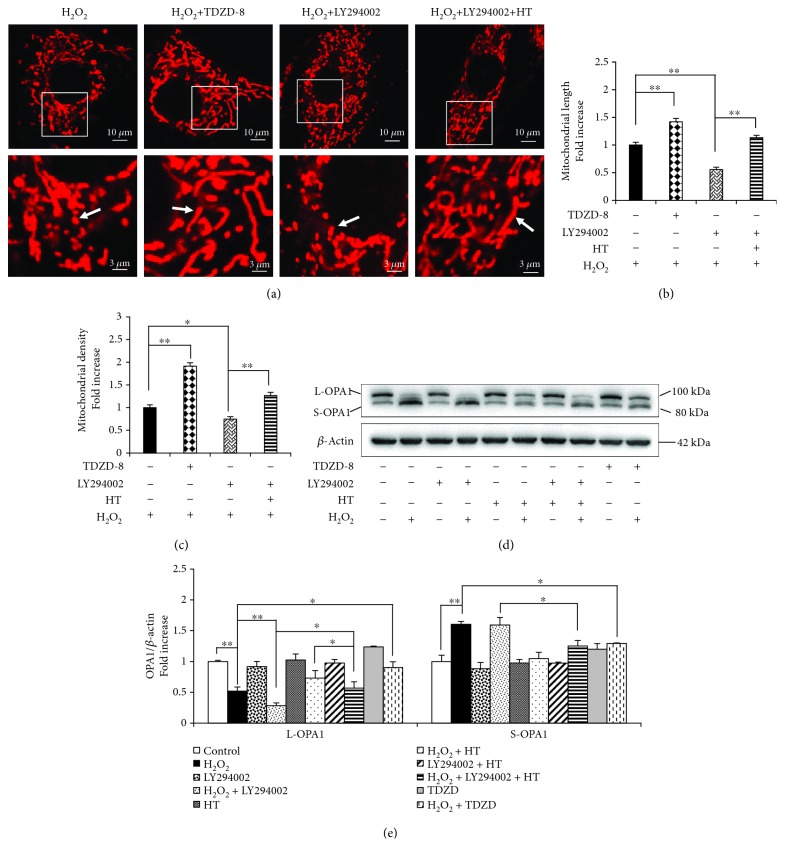
HT protects the mitochondrial morphology and function through the AKT-GSK3*β* signaling pathway. (a–c) Representative images showing the mitochondrial morphology, length, and density in the indicated groups. Error bars indicate SD (*n* = 3); scale bars, 10 *μ*m. (d) Representative immunoreactive bands of OPA1 in the osteoblasts treated with H_2_O_2_. (e) Quantification of the immunoreactive bands of OPA1 relative to that of *β*-actin. Error bars indicate SD (*n* = 6). Full-length blots are presented in Supplementary Figure 1. ^∗^ *p* < 0.05 and ^∗∗^ *p* < 0.0001, as determined by comparing selected pairs (each test was repeated three times).

HT activates AKT and induces the phosphorylation of some related kinases, and this may play a pivotal role in protecting mitochondrial membrane systems [32, 33]. However, it is unclear whether the AKT-GSK3*β* signaling pathway regulates mitochondrial morphology and function. We found that TDZD-8 preserved the mitochondrial morphology in the presence of H_2_O_2_ but disrupted it in the presence of LY294002. Moreover, we found that HT reversed mitochondrial morphological abnormalities and dysfunction by maintaining the mitochondrial length and shape that were impaired by LY294002 and H_2_O_2_ cotreatment (Figures 6(a)–6(c)). These results confirm the involvement of the AKT-GSK3*β* signaling pathway in regulating mitochondrial morphology.

OPA1 plays an essential role in mitochondrial fusion. AKT inhibition by LY294002 abolished the protective effect of HT by inhibiting OPA1 cleavage (Figures 6(d) and 6(e)). These results indicate that active AKT is required for promoting mitochondrial fusion and that HT prevents mitochondrial fragmentation through active AKT. In addition, TDZD-8 considerably inhibited H_2_O_2_-induced OPA1 cleavage (Figures 6(d) and 6(e)). Together, these results provide direct evidence that HT prevents osteoblast apoptosis and mitochondrial dysfunction by preventing OPA1 cleavage and by activating the AKT-GSK3*β* signaling pathway.

## 4. Discussion

OS-induced osteoblast apoptosis is critical for osteoporosis development; however, mechanism underlying this process has not been thoroughly elucidated to date. Accumulating evidence has shown that an imbalance in mitochondrial dynamics is closely associated with OS [14, 34]. However, few studies have investigated this association in OS-induced osteoblast apoptosis. The present study is the first to show that OPA1 cleavage contributes to OS-induced mitochondrial dysfunction and osteoblast apoptosis. The AKT-GSK3*β* signaling pathway may regulate OPA1 cleavage. Importantly, we observed that HT promoted osteoblast survival by preventing OS-induced OPA1 cleavage and by activating the AKT-GSK3*β* signaling pathway. Together, our data indicate that HT is a beneficial agent for preventing osteoporosis by enhancing osteoblast survival.

The mitochondria are the major energy-producing organelles that play a significant role in determining cell survival and death [35–37]. In osteoblasts, the mitochondria are specialized for calcium transport and are involved in the calcification of the extracellular matrix [38]. Moreover, the mitochondrial respiratory chain complexes regulate osteogenic differentiation [39]. Therefore, mitochondrial dysfunction in osteoblasts is suggested to directly and indirectly play a role in osteoporosis. In the present study, we observed that H_2_O_2_ treatment decreased the viability and increased the apoptosis of the MC3T3-E1 cells (Figure 1), which was consistent with that reported in our previous study [11]. H_2_O_2_ significantly increased mtROS production and considerably decreased MMP, respiratory chain complex activity, and ATP production. Previously, we found that an antioxidant ethylenediamine chloride (EUK-134) significantly decreased H_2_O_2_-induced osteoblast apoptosis and mitochondrial dysfunction [11]. These findings strongly support the pivotal role of mtROS in mediating mitochondrial dysfunction and in promoting osteoblast apoptosis [11, 36].

The balance of mitochondrial dynamics is critical for maintaining mitochondrial function and energy generation and for preventing apoptosis [34, 40, 41]. OPA1 is located in the mitochondrial inner membrane and regulates mitochondrial fusion. Moreover, OPA1 is suggested to play direct roles in preventing apoptosis [42]. Several studies have shown that a decrease in mitochondrial ATP levels due to apoptosis induction or MMP loss is a crucial stimulus that regulates OPA1 processing [13, 16, 17]. L-OPA1 undergoes further processing in the matrix to produce S-OPA1. OPA1 knockdown-induced mitochondrial fragmentation is rescued by L-OPA1 expression rather than by S-OPA1 expression [43]. Preservation of a stable L-OPA1 pool in the mitochondrial inner membrane helps in maintaining sufficient mitochondrial content to preserve cell viability [13]. The results of the present study showed that H_2_O_2_ promoted the rapid cleavage of L-OPA1 into S-OPA1 in osteoblasts, leading to the loss of fusion-active L-OPA1, which exacerbated mitochondrial fragmentation and subsequent osteoblast apoptosis (Figure 3). OPA1 also regulates mitochondrial ATP production, especially for the assembly of ATP synthase [18]. Zhang et al. reported that *β*-cells lacking OPA1 maintained normal mitochondrial DNA copy numbers; however, the amount and activity of electron transport chain complex IV were significantly decreased in these cells, leading to impaired glucose-stimulated ATP production and insulin secretion [18]. Therefore, we speculated that ROS promotes osteoblast apoptosis by modulating OPA1. As expected, transfection of the MC3T3-E1 cells with the OPA1 siRNA aggravated H_2_O_2_-induced apoptosis and mitochondrial morphological abnormalities. These results indicate a close association between mitochondrial dysfunction-associated OPA1 cleavage and osteoblast apoptosis. However, detailed regulatory mechanisms underlying this could not be explored. Therefore, additional studies are needed to investigate the role of abnormal OPA1 cleavage in osteoblast apoptosis. However, our results suggest that novel therapeutic strategies, including the maintenance of mitochondrial function and prevention of OPA1 cleavage, can prevent osteoblast apoptosis in osteoporotic patients.

AKT initiates downstream pathways that inhibit apoptosis [44]. Activated AKT exerts various biological effects by promoting the phosphorylation of downstream substrates such as GSK3*β*. Moreover, p-AKT inactivates GSK3*β* by phosphorylating it, thus preventing the opening of mitochondrial permeability transition pores, which are important master regulators of cell death [45]. In the present study, the S-OPA1 level increased and the L-OPA1 level decreased in the MC3T3-E1 cells treated with the same dose of H_2_O_2_ after AKT inactivation (by pretreatment with LY294002). Moreover, inhibition of p-GSK3*β* activity (by pretreatment with TDZD-8), followed by treatment with the same dose of H_2_O_2_, increased the L-OPA1 level and decreased the S-OPA1 level. These results confirm that OS induces OPA1 cleavage by directly modulating the AKT-GSK3*β* signaling pathway. In previous studies describing AKT-dependent regulation of NF*κ*B [46] and the ability of AKT-mTOR-NF*κ*B-OPA1 signaling pathway to influence mitochondrial function [22], GSK3*β* and NF*κ*B play crucial roles in regulating mitochondrial morphology and dynamics [47]. Therefore, further studies are required to explore whether these signaling pathways are associated with OPA1 cleavage during osteoblast apoptosis.

HT and its derivatives are the major polyphenolic compounds in olive oil and have several pharmacological properties, including antioxidant and anti-inflammatory properties [48, 49]. Kitsati et al. reported that HT strongly protects Jurkat cells against H_2_O_2_-induced apoptosis [24]. Moreover, high doses of HT induce the apoptosis of papillary and follicular thyroid cancer cells [50]. In our study, we found that a low dose of HT efficiently prevented OS-induced apoptosis of the MC3T3-E1 cells. Moreover, we observed that these cytoprotective effects of HT were similar to those of NAC (the classical free radical scavenger). Thus, the results of the present study confirm the antioxidant property of HT. In addition, our results indicate that HT increases mitochondrial function, suppresses mtROS production, and restores mitochondrial dynamics imbalance. A recent study has shown that HT exerts beneficial effects against mitochondrial damage under pathological conditions [13]. In the present study, HT directly reduced OS-induced rapid cleavage of L-OPA1 to S-OPA1 in osteoblasts. Interestingly, OPA1 knockdown abolished the antiapoptotic and mitochondria-protective effects of HT, which increased OS-induced apoptosis and associated mitochondrial abnormalities. These results suggest that an OPA1-dependent mitochondrial pathway is the direct target of HT for preventing osteoblast apoptosis.

We also found that HT significantly enhanced the phosphorylation of AKT (Ser473) and GSK3*β* (Ser9). However, the antiapoptotic effect of HT was significantly abolished by LY294002 and inhibition of GSK3*β* significantly suppressed H_2_O_2_-induced osteoblast apoptosis. One study suggested that HT exerted antiapoptotic and cardioprotective effects against myocardial I/R injury and that these effects were associated with the activation of the AKTGSK3*β* signaling pathway in the myocardium [51]. Thus, the results of the present study and of previous studies suggest that HT exerts a protective effect against osteoblast apoptosis through the AKT-GSK3*β* signaling pathway, which regulates OS-induced OPA1 cleavage.

In the present study, we found that HT effectively attenuated OS-induced osteoblast apoptosis and determined the underlying mitochondrial mechanism. However, this study has several limitations. First, detailed mechanisms of the AKT-GSK3*β* signaling pathway and OPA1 in OS-induced osteoblast apoptosis could not be determined in the present study. Therefore, additional studies are needed to explore these mechanisms. Second, the present study did not involve additional cell lines, primary cultured osteoblasts, and *in vivo* models. Therefore, additional studies should be performed using these experimental models to confirm mitochondrial mechanisms underlying OS-induced cell apoptosis and to verify the effect of HT.

**Figure 7:**
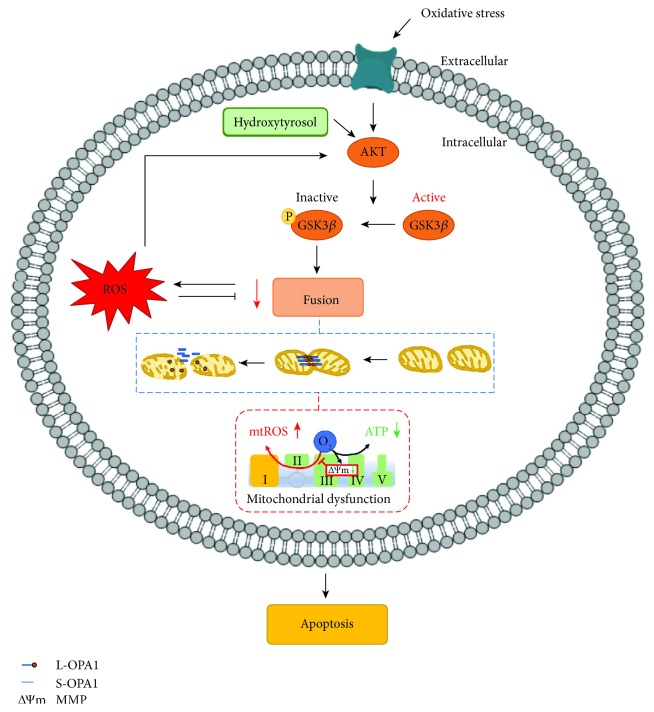
HT regulates OS-induced mitochondrial dysfunction and osteoblast apoptosis. In the MC3T3-E1 cells, HT treatment affected OPA1 cleavage, thus preventing mitochondrial dysfunction. Moreover, these changes were found to be dependent on the AKT-GSK3*β* signaling pathway.

To the best of our knowledge, the present study is the first to show that HT prevents OS-induced osteoblast apoptosis and maintains mitochondrial function in cultured osteoblasts by stimulating mitochondrial fusion. Moreover, our results indicate that OS-induced osteoblast apoptosis is regulated by OPA1 cleavage, which induces mitochondrial dysfunction. Furthermore, our results indicate that these changes at least in part depend on the AKT-GSK3*β* signaling pathway (Figure 7). However, additional studies are needed to identify potential new targets and effective nutrients for developing new strategies for preventing and treating osteoporosis.

## Data Availability

The data used to support the findings of this study are available from the corresponding author upon request.

## Supplementary Materials

Supplementary Materials Supplementary Figure 1: uncropped full-length pictures of Western blotting membranes. Uncropped full-length pictures of Western blotting membranes presented in the main figures. Membranes were often cut to enable blotting for multiple antibodies.
