# Supplementary material for: AKT-GSK3β Signaling Pathway Regulates Mitochondrial Dysfunction-Associated OPA1 Cleavage Contributing to Osteoblast Apoptosis: Preventative Effects of Hydroxytyrosol
Source: Oxid Med Cell Longev. 2019 Jun 2;2019:4101738. doi: 10.1155/2019/4101738 (PMC6589274; doi:10.1155/2019/4101738)
Supplement: Supplementary Materials — Supplementary Figure 1: uncropped full-length pictures of Western blotting membranes. Uncropped full-length pictures of Western blotting membranes presented in the main figures. Membranes were often cut to enable blotting for multiple antibodies. [file 4101738.f1.docx]

**Supplementary Figure Legends**

**Supplementary Figure 1**


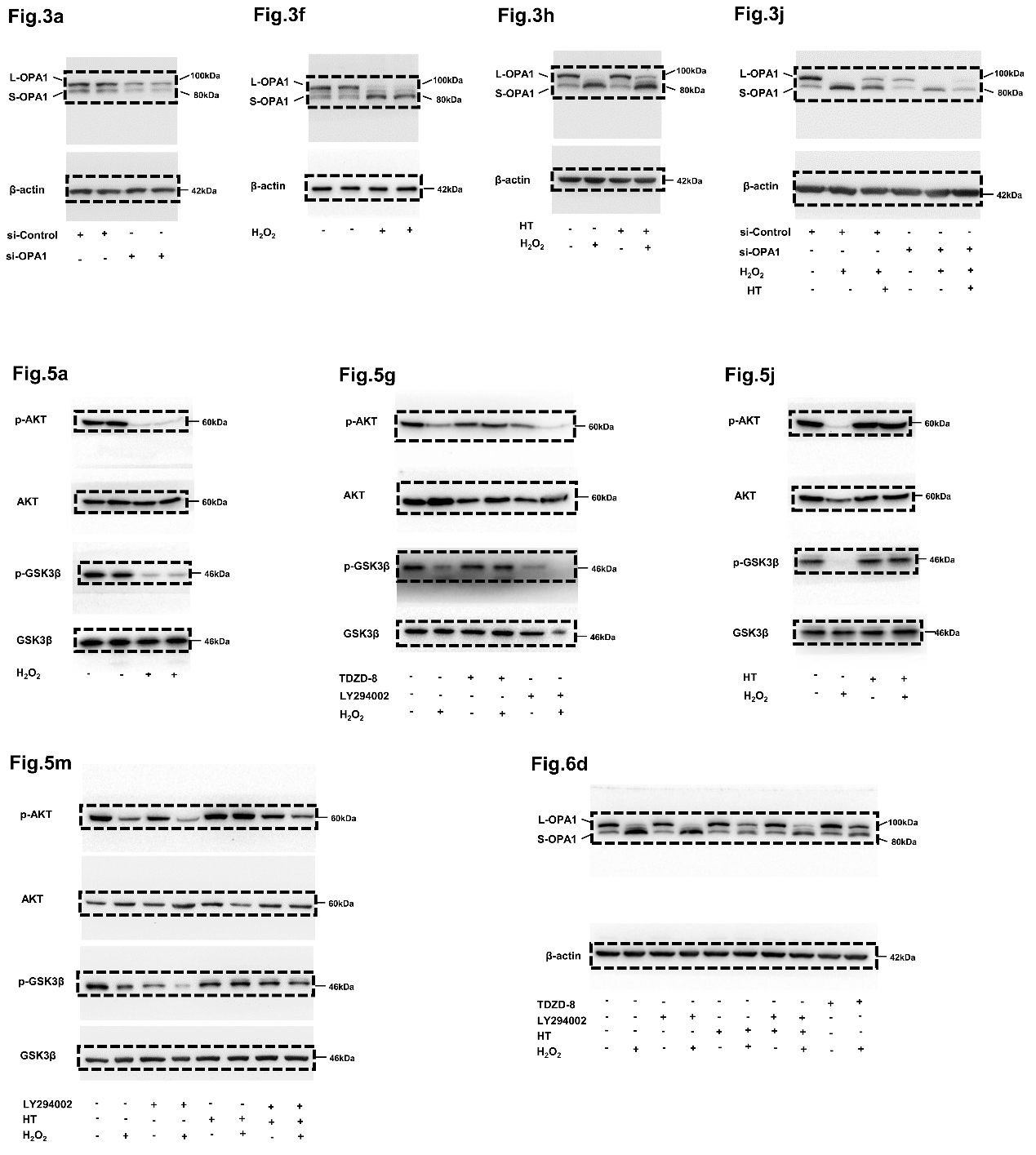


**Supplementary Figure 1 Uncropped full-length pictures of Western blotting membranes.** Uncropped full-length pictures of Western blotting membranes presented in the main figures. Membranes were often cut to enable blotting for multiple antibodies.
